# First person – Kerry Roby

**DOI:** 10.1242/bio.059212

**Published:** 2022-02-21

## Abstract

First Person is a series of interviews with the first authors of a selection of papers published in Biology Open, helping early-career researchers promote themselves alongside their papers. Kerry Roby is first author on ‘ Loss of p19Arf Promotes Fibroblast Survival During Leucine Deprivation’, published in BiO. Kerry conducted the research described in this article while a postgraduate student in Sandra Ryeom's lab at the Perlman School of Medicine, University of Pennsylvania. He is now a Postdoc in the lab of Joseph McCarty at the MD Anderson Cancer Center, University of Texas, investigating the ‘normal’ cell populations of the tumor microenvironment, and how their manipulation promotes tumorigenesis.


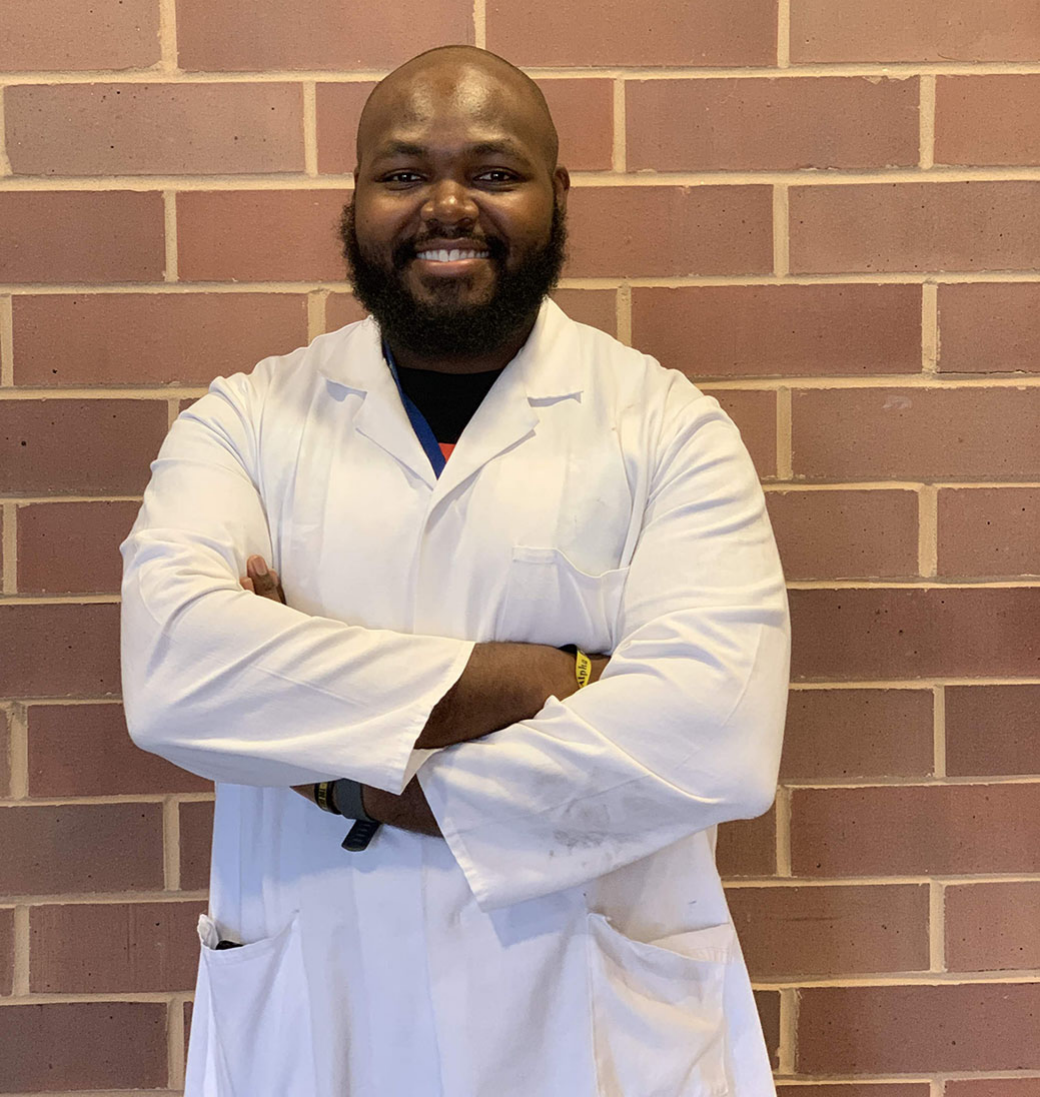


**Kerry Roby**

**What is your scientific background and the general focus of your lab?**

My scientific background is cancer biology. During my graduate studies in the Ryeom lab, the general focus was the role of endothelial and stromal cells in tumor progression and metastasis.

**How would you explain the main findings of your paper to non-scientific family and friends?**

In cancer, normal cells and cancer cells are exposed to stresses including the limited availability of nutrients such as amino acids. Normal cells, such as fibroblasts, are often altered in this environment. To slow tumor advancement, the body activates tumor suppressors including the p19 Alternate reading frame protein (p19Arf). The response to stress by fibroblasts and the effects of p19Arf in fibroblasts are not well understood. Our paper studied how the loss of p19Arf in fibroblasts affected their response to prolonged absence of leucine. We showed that normal fibroblasts exposed to prolonged leucine deprivation increased the production of p19Arf resulting in cell death, and the absence of p19Arf promoted fibroblast survival.

**What are the potential implications of these results for your field of research?**

Our results identify a new role for the p19Arf tumor suppressor in stromal cells in response to amino acid deprivation, a common stress of the tumor microenvironment. Our results indicate a role for p19Arf in ‘normal’ cells of the tumor microenvironment along with tumor cells.

**Figure:**
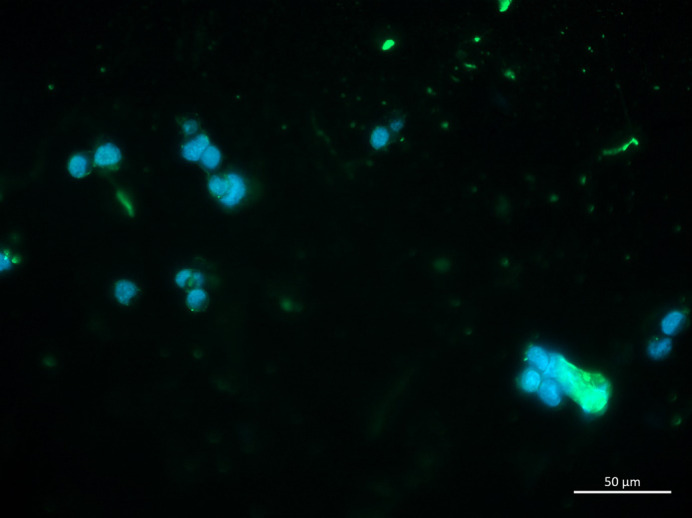
Lewis Lung Carcinoma (LLC) tumoroids generated in the presence of *p19Arf*^−/−^ adult lung fibroblasts.

**What has surprised you the most while conducting your research?**

My biggest surprise was the ability to generate 3D tumoroids and expose them to stresses in an *in vitro* setting that more closely recapitulates the *in vivo* setting.

**What, in your opinion, are some of the greatest achievements in your field and how has this influenced your research?**

The recent studies identifying subsets of stromal cells in the tumor microenvironment further reveals the heterogeneity of these cells and the complexity of tumors.

**What changes do you think could improve the professional lives of early-career scientists?**

The professional lives of early-career scientists can be improved by more mentoring and specifically connecting early-career scientists with mentors from similar backgrounds. With the ease of zoom, mentors no longer need to be at the same institution and these relationships are critical to learn how to navigate a biomedical career and share expectations of junior scientists and how best to advance.

With the ease of zoom, mentors no longer need to be at the same institution...

**What's next for you?**

I've transitioned to a postdoc in the Neurosurgery Department at the University of Texas MD Anderson Cancer Center.
